# Supplementary material for: Flow cytometry multiplexed method for the detection of neutralizing human antibodies to the native SARS‐CoV‐2 spike protein
Source: EMBO Mol Med. 2021 Feb 17;13(3):e13549. doi: 10.15252/emmm.202013549 (PMC7933943; doi:10.15252/emmm.202013549)
Supplement: Supplementary file 1 — Expanded View Figures PDF [file EMMM-13-e13549-s001.pdf]

Expanded View Figures

Figure EV1. Filtering and multivariate normal function adjustment of cytometry data. RyC70 is a seronegative sample; RyC58 a seropositive one.

A, B First gating on the SSC.A and FSC.A channels using a t-mixture model with Box-Cox transformation for clustering. A 2D plot of the signal from the cells in the SSC.A and FSC.A channels (gray, red, and green dots) is shown. In (A) a single cluster is adjusted (cells included in the cluster in red, not selected in gray), while in (B) two clusters are adjusted (cells in red for the first cluster and green for the second).

C Over the cells selected in the first gating, a second clustering is done with the same method using channels FL7.A and FL2.A. Selected cells are marked in red, and unselected cells are represented in gray.

D Adjustment of the multivariate normal distribution over the signal from the FL7.A and FL2.A channels from the selected cells after the first and second gating. 1, contour of the density function fitted to the data. 2, density plot of the signal in channel FL7.A. 3, density plot of the signal in channel FL2.A. 4, 2D plot of signal from cells in channels FL7.A and FL2.A.

© 2021 The Authors

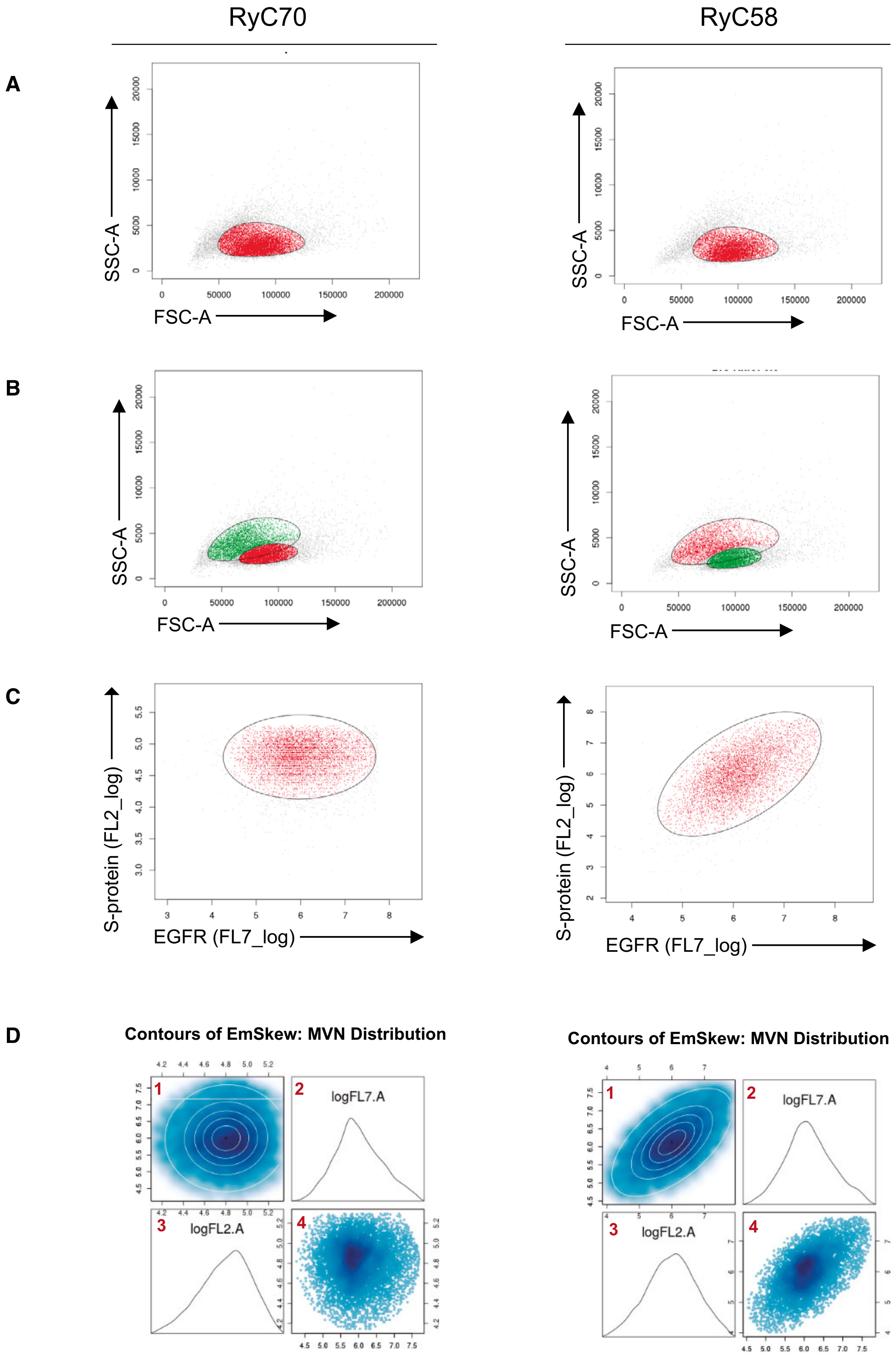
RyC70
RyC58
A
B
C
D
SSC-A
FSC-A
S-protein (FL2_log)
EGFR (FL7_log)
Contours of EmSkew: MVN Distribution
logFL7.A
logFL2.A

Figure EV1.

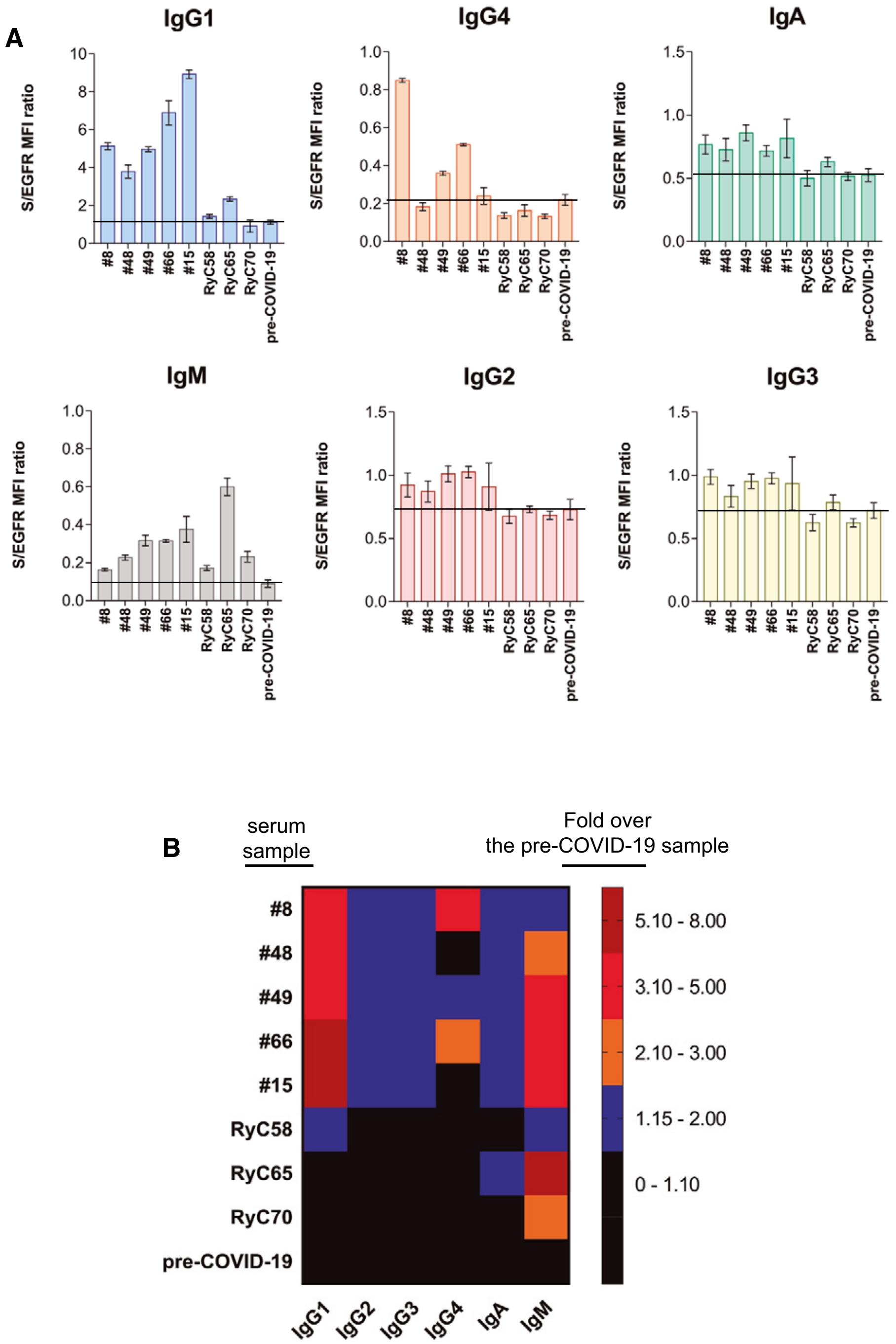

Figure EV2. Flow cytometry of Jurkat-S cells can be used in multiplexed methods to detect simultaneously the expression of anti-S antibodies of all subclasses.

A Detection of anti-S immunoglobulins of the indicated subclasses in a multiplexed method of flow cytometry in the serum samples indicated at 1:50 dilution. Data represent the mean ± SD of triplicated datasets.

B Rainbow heatmap plot of the anti-S protein antibody data shown in (A), normalized as fold value of the S/egfr MFI ratio in each sample divided by the mean S/egfr MFI ratio of the pre-COVID-19 serum.

 © 2021 The Authors
