# Supplementary material for: Flow cytometry multiplexed method for the detection of neutralizing human antibodies to the native SARS‐CoV‐2 spike protein
Source: EMBO Mol Med. 2021 Feb 17;13(3):e13549. doi: 10.15252/emmm.202013549 (PMC7933943; doi:10.15252/emmm.202013549)
Supplement: Supplementary file 3 — Table EV2 [file EMMM-13-e13549-s003.docx]

**Legend:**

**Patient code**: it is a random internal number

**Age:** in years

**Sex:** M, male; F, female

**Clinical Score:** clinical classification according to the following parameters: Asymptomatic, no symptoms; Mild, 3 or more of the following symptoms: non-productive cough, hyperthermia, headache, odynophagia, dyspnea, asthenia, myalgia, ageusia, anosmia, cutaneous involvement; Moderate, 3 or more of the above symptoms plus gastrointestinal symptoms,  or  more than 3 of the above for 7 or more days; Moderate-Severe, 3 or more of the above symptoms plus pneumonia; Severe, pneumonia  requiring hospitalization and intubation.

**PCR Test:** If the volunteers had been analyzed by PCR for infection by SARS-CoV2 or not.

**Result of the PCR Test**: Positive or Negative.

**VIRCLIA ELISA (IgM and IgA) Test**: Positive, Negative or an Unclear result.

**Anti-S Flow Cytometry (MFI):** Mean fluorescence Intensity resulting of staining Jurkat-S cells with a 1:50 dilution of the sera and a PE-labeled anti-human IgG1 antibody. Clearly positive results in green, unclear in yellow, negative in pink.

**Anti-S Flow Cyotometry (ratio):** Ratio of the MFI for anti-S antibodies in Jurkat-S cells divided by the MFI with an anti-EGFR antibody used to normalize. Clearly positive results in green, unclear in yellow, negative in pink.

**Score:** Score defined according to the algorithms described in Materials and Methods. A Score >0.024 is considered positive.
