# Supplementary material for: Flow cytometry multiplexed method for the detection of neutralizing human antibodies to the native SARS‐CoV‐2 spike protein
Source: EMBO Mol Med. 2021 Feb 17;13(3):e13549. doi: 10.15252/emmm.202013549 (PMC7933943; doi:10.15252/emmm.202013549)
Supplement: Supplementary file 5 — Table EV4 [file EMMM-13-e13549-s005.docx]

**Legend:**

**Patient code**: it is a random internal number

**Age:** in years

**Sex:** M, male; F, female

**Clinical Score:** clinical classification according to the following parameters: Asymptomatic, no symptoms; Mild, 3 or more of the following symptoms: non-productive cough, hyperthermia, headache, odynophagia, dyspnea, asthenia, myalgia, ageusia, anosmia, cutaneous involvement; Moderate, 3 or more of the above symptoms plus gastrointestinal symptoms,  or  more than 3 of the above for 7 or more days; Moderate-Severe, 3 or more of the above symptoms plus pneumonia; Severe, pneumonia  requiring hospitalization and intubation.

**PCR Test:** If the volunteers had been analyzed by PCR for infection by SARS-CoV2 or not.

**Result of the PCR Test**: Positive or Negative.

**ELISA Test**: Positive or Negative. Test carried out by the National Health System of undisclosed origin.

**Result of the FC Jurkat-S test according to the Score value:** Positive or Negative.
